# Seeking Solitude After Being Ostracized: A Replication and Beyond

**DOI:** 10.1177/0146167220928238

**Published:** 2020-06-09

**Authors:** Dongning Ren, Eric D. Wesselmann, Ilja van Beest

**Affiliations:** 1 Department of Social Psychology, Tilburg University, The Netherlands; 2 Illinois State University, Normal, USA

**Keywords:** solitude, ostracism, exclusion, rejection, extraversion

## Abstract

Individuals may respond to ostracism by either behaving prosocially or antisocially. A recent paper provides evidence for a third response: solitude seeking, suggesting that ostracized individuals may ironically engage in self-perpetuating behaviors which exacerbate social isolation. To examine this counterintuitive response to ostracism, we conceptually replicated the original paper in three studies (*N* = 1,118). Ostracism experiences were associated with preference for solitude across four samples (Study 1), and being ostracized increased participants’ desires for solitude (Studies 2 and 3). Extending beyond the original paper, we demonstrated that only the experience of being ostracized, but not ostracizing others or the feeling of conspicuousness, triggered the desire for solitude. Diverging from the original paper, trait extraversion did not moderate the effect of ostracism on solitude desires. Taken together, the current research provides additional and stronger empirical evidence that solitude seeking is a common response to ostracism.

Ostracism^1^—being ignored and excluded—causes psychological pain and threatens fundamental psychological needs (i.e., belonging, self-esteem, meaningful existence, and control; Williams, 2009). Ostracized individuals often respond to this stressful event with one of two behavioral patterns: antisocial behaviors or prosocial behaviors. For example, several experimental studies have found that ostracized participants are more likely than included participants to respond aggressively, whether they are aggressing against the ostracizer or an innocent third party (e.g., Chow et al., 2008; Twenge et al., 2001; Warburton et al., 2006). Other experimental studies have found that ostracized participants respond more prosocially than included participants, defined as increased cooperation (Williams & Sommer, 1997; but see Walasek et al., 2019), increased interest in new groups and re-affiliation strategies (Maner et al., 2007; Mead et al., 2010), and increased susceptibility to social influence tactics (e.g., conformity and compliance; Carter-Sowell et al., 2008; Williams et al., 2000).

A third response has recently been introduced to the literature: *solitude seeking* (Wesselmann et al., 2014). However, to date, only one published manuscript has examined and provided empirical support to this response to ostracism (Ren et al., 2016). It is important to put this prediction to additional empirical testing for two reasons. On one hand, this prediction seems contradictory with the main theories in ostracism research. Belonging is a basic human need (Baumeister & Leary, 1995; Ryan & Deci, 2000). People with an unsatisfied belonging need experience a wide range of harmful outcomes ranging from negative affect and impaired cognitive abilities, to depressive symptoms, to early mortality (Hawkley & Cacioppo, 2010; Riva et al., 2017; Twenge et al., 2003). Given that belonging is essential for physical and psychological well-being, it seems only reasonable that ostracized individuals spare no effort in “goal-oriented behaviors” that satisfy their need to belong (Baumeister & Leary, 1995, p. 498), such as establishing new connections (Maner et al., 2007). Second, seeking further isolation after being ostracized has negative consequences for one’s health and well-being. Ostracized individuals are temporarily socially disconnected; by choosing to move further away from the social world, they deny themselves any potential opportunities to reestablish connections. This self-perpetuating behavior of ostracized individuals suggest the possibility that an ostracism episode may trigger a downward spiral into loneliness and social isolation (Williams, 2009).

We set out to examine the effect of ostracism on solitude seeking. To do so, we conducted three conceptual replication studies of Ren et al. (2016), focusing on eliminating alternative explanations and exploring potential mediators and the moderating role of extraversion.

## Ostracism Stimulates the Desire for Solitude

Why would ostracized individuals seek solitude? It has been theorized that, in response to threatening social situations, people may move away from social situations as a coping strategy (Van Kleef et al., 2010). This response is argued to be promoted by an integrated set of cognitive (e.g., “I am undesirable.”), emotional (e.g., shame), and biological changes (e.g., increases in inflammation; Slavich et al., 2010). By retreating into solitude, one may minimize the risks of additional social injury (Richman & Leary, 2009; Sunami et al., 2019a; Wesselmann et al., 2014).

Empirical work provides suggestive evidence that ostracized people are motivated to seek shelter in solitude. For example, rejected children are less engaged in classrooms and express a desire to avoid school (Buhs & Ladd, 2001). The experience or anticipation of negative interpersonal events (e.g., having conflicts with one’s romantic partner) is considered as the most common reason why someone would prefer to spend time alone (Wesselmann et al., 2014). At least half of a million Japanese suffer from Hikikomori, a psychological condition among bullied or excluded people who lock themselves in their own houses for months and years (Furlong, 2008; Kaneko, 2006). In experimental studies, compared with included participants, ostracized participants reported higher intentions to disengage from social situations (Pfundmair et al., 2015), devalued their subsequent interaction partners (Sommer & Bernieri, 2015), showed an increased liking of physical spaces that hinder social interaction (Meagher & Marsh, 2017), judged other people’s eye gaze to be averted—a signal that others are unapproachable (Syrjämäki et al., 2018), and were more prevention-oriented, reflecting their desire to avoid being rejected again (Park & Baumeister, 2015).

One recent report (Ren et al., 2016) provided direct empirical evidence that ostracism increases solitude desires. In Study 1 (correlational), people who reported having higher levels of ostracism experience also reported stronger preference for solitude. In Studies 2 to 4 (experimental), participants’ ostracism experience was manipulated through either a virtual ball-tossing game which has been widely adopted in ostracism experimental research (Cyberball; Williams et al., 2000) or a face-to-face role-play activity (“O-train”; Zadro et al., 2005). Afterward, participants reported their preference for being alone in a subsequent activity. Consistent across three experiments, ostracized participants indicated a stronger desire to be alone than included participants. This effect was also found to be more prominent among participants who score low in extraversion (Study 4).

This set of studies lends support to the third solitude option, but they are the only studies that directly examined the effect of ostracism on solitude. Moreover, these studies were limited in several ways. First, the reported correlation (*r* = .26) between ostracism experiences and preference for solitude may be unreliable. This correlation was estimated based on 100 participants (original Study 1), but 250 is usually recommended to estimate stable correlations (Schönbrodt & Perugini, 2013).

Second, the effect of ostracism on solitude was solely studied from the perspective of targets. Despite that each ostracism episode involves both the target—the one who is being ostracized, and the source—the one(s) who are ostracizing, researchers have long focused on targets’ perspectives but not sources (Zadro et al., 2017). As a result, to date we have a limited understanding of how sources experience or react to ostracism events. Similarly, the original paper suffers from the same limitation, failing to offer any insight into sources’ preference for solitude after ostracism. It is possible that the sources of ostracism experience distress while excluding others (Chen et al., 2014; Ciarocco et al., 2001; Poulsen & Kashy, 2012) and consequently seek more solitude. Said another way, people who are involved in ostracism events may seek more solitude than those who were not, regardless whether they are targets or sources. To test this possibility, both perspectives should be examined simultaneously.

Third, a possible confounding variable was present in the original set of experiments. In these experiments, participants were either included or ostracized. This is a commonly used design in the social exclusion literature, but one confound, as identified by past research, is the feeling of conspicuousness (Williams et al., 2000). Specifically, included participants were not likely to feel conspicuous because they were treated as an equal member, while ostracized participants were likely to feel conspicuous or self-aware because they received much less attention than the rest of the group. We consider this confounding variable to be important here, because feeling conspicuous, or self-conscious is frequently associated with shyness, social anxiety, and social withdrawal (Alden et al., 1992; Brown et al., 2007; Cheek & Melchior, 1990; Fenigstein et al., 1975), leaving it possible that ostracized individuals may have sought solitude simply because they felt conspicuous. To rule out this alternative explanation, researchers (Williams et al., 2000) have recommended adding an overinclusion condition—which does not lead to feelings of ostracism but may still evoke conspicuousness due to excessive attention—to the typical inclusion versus ostracism design.

## Current Research

We conducted three studies with each study focusing on addressing a separate limitation identified in the original paper. We first estimated the association between ostracism experiences and preference for solitude with a relatively large sample in Study 1. We then experimentally tested the effect of ostracism on participants’ desire for solitude in Studies 2 and 3. Extending beyond the original paper, we examined both perspectives of targets and sources simultaneously in Study 2 and improved the original experimental design by adding an overinclusion condition to minimize a confounding effect in Study 3. In addition, Study 3 tested whether trait extraversion moderates the effect of ostracism on solitude; Studies 2 and 3 both explored potential mediators of this effect.

All research materials, data, and analysis scripts are available at the Open Science Framework: https://osf.io/9rvb3/

## Study 1

This study specifically addresses the limitation of sample size in Ren et al. (2016) Study 1. Whereas the original study collected a small online sample from U.S. MTurk users; here, we drew larger samples from college students in Europe.

### Method

#### Participants

First year psychology students from a university in the Netherlands participated in a prescreening survey administered at the beginning of a semester for course credits. We combined datasets that were available to us to maximize sample size. These datasets were collected using either Dutch or English and during 2016 and 2017 (initial *N* = 658). Seven participants were excluded for not completing our study variables, leaving the final sample size 651 (Table 1). Of this sample, 68% were from the Netherlands, 17% were from Germany, 10% were from other European countries, and 5% were from non-European countries, have dual nationality, or did not report their nationality.

**Table 1.** Sample Characteristics in Study 1.

Samples	Year	Language	N	Gender^a^%			Age M (SD)
				Male	Female	Other	
1	2016	Dutch	187	18.7	80.2	0.5	19.54 (2.68)
2	2016	English	80	20.0	77.5	0.0	20.51 (2.69)
3	2017	Dutch	256	23.4	76.2	0.4	19.42 (2.00)
4	2017	English	128	23.4	76.6	0.0	20.29 (2.70)
Combined			651	21.7	77.6	0.3	19.76 (2.47)

*Note.* In total, three participants did not report gender or age.

^a^ In Study 1, gender was measured using three options: male, female, and other/no answer. In Studies 2 and 3, the first two options were provided.

#### Procedure and materials

Data collection procedure was identical for all samples. Participants were brought into the laboratory and assigned to individual cubicles to complete a survey packet consisting of several unrelated questionnaires on a computer. The survey took about 1 hr to complete, and measures of ostracism experiences and preference for solitude were embedded in this survey. These two measures were the variables of interest for this research, and thus we will not discuss any of the other measures further. Both measures were originally in English; the Dutch versions of both measures went through translation and back translation procedure (Brislin, 1970; see Supplemental Materials for the translated versions).

#### Ostracism experience

We used the same scale as in Ren et al. (2016), the Ostracism Experience Scale (Carter-Sowell, 2010; Gilman et al., 2013). To our knowledge, this is the only scale in the literature to measure participants’ *general* ostracism experiences in daily life. The scale included eight items rated on a 7-point scale (1 = *hardly ever*, 7 = *almost always*) regarding how often each scenario happens (e.g., “In general, others do not look at me when I am in their presence.”). We created a single index by averaging all items together such that higher numbers reflected more ostracism experiences (α = .90).

#### Preference for solitude

Ren et al. (2016) used the Preference for Solitude Scale (Burger, 1995) and an adapted version of the scale to measure participants’ preference for solitude. These two scales were found to be highly correlated with each other, and both were correlated with ostracism experiences similarly. For the brevity of the prescreening survey, here we only included the adapted version. The adapted version used similar items from the original scale (16 items; e.g., “I need time alone each day.”) but replaced the original forced-choice format with a 7-point scale (1 = *not at all*, 7 = *very much*). Items were reverse-coded when necessary and averaged to form an index of general preference for solitude (α = .90).

### Results

**Figure 1.**
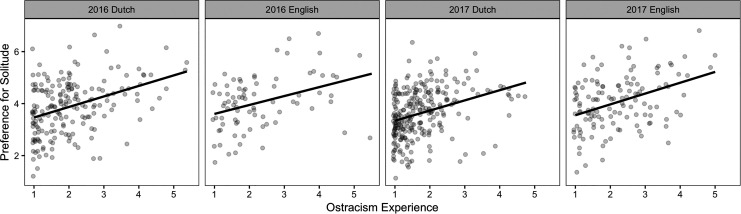
The association between ostracism experiences and preference for solitude in Study 1.

To estimate the association between ostracism experiences and preference for solitude, and to test whether the association is robust across samples, we estimated a regression model. This model included participants’ ostracism experience score (mean-centered), the year of the sample collected (2016 or 2017), the language of the survey administrated (Dutch or English), and their interaction terms as predictors; participants’ preference for solitude as the outcome variable. The main effect of ostracism experiences was significant (*B* = 0.40, confidence interval [CI] = [0.27, 0.54],^2^ *p* < .001). No other effects were significant (|*B*|s < 0.16, *p*s > .13; Figure 1).

### Discussion

Study 1 serves as a conceptual replication of the original correlational study (Ren et al., 2016, Study 1), using a different data collection procedure (laboratory vs. online) and samples drawn from a different population (College students in Europe vs. U.S. MTurk users). Despite these deviations, we replicated the positive association between ostracism experiences and solitude preferences. This association was robust across four independent samples; neither the year the data were collected nor the language of the measures significantly affected this correlation.

Next we turn our attention to replicating the causal effect of ostracism on solitude. In the subsequent two studies, we manipulated participants’ ostracism experiences using different paradigms and measured their desire for solitude afterwards.

## Study 2

The goal of this study was to test the effect of ostracism on solitude seeking among targets and sources simultaneously. One challenge to experimentally studying both perspectives simultaneously is the lack of suitable paradigms (Zadro et al., 2017). Most available paradigms manipulate ostracism experiences from one perspective, focusing on either the targets (e.g., Cyberball; Williams et al., 2000) or the sources (e.g., a modified Cyberball, Wesselmann et al., 2013). One of the only paradigms that manipulates ostracism from both perspectives is O-train (Zadro et al., 2005), in which participants form triads and in each triad two participants (source) are instructed to either include or ostracize the third (target). Although this paradigm was used in one of the original experiments (Ren et al., 2016, Study 3), the authors only analyzed the responses from the targets and did not analyze or report the responses from the sources. Here, we improved the analytic approach to examine targets and sources simultaneously. To increase statistical power and sample heterogeneity (Curran & Hussong, 2009), we combined the original sample reported in Ren et al. (2016) with three additional samples that we collected using the same procedure and materials of the original experiment.^3^ We expected an interaction effect between participants’ ostracism experience (inclusion vs. ostracism) and their role in that experience (target vs. source), such that ostracism only motivate solitude seeking among targets but not sources.

### Method

#### Participants

The combined sample consists of 79 O-train triads (40 from the original study, 39 newly collected) with 41 randomly assigned to the inclusion condition and 38 to the ostracism condition (Table 2).

**Table 2.** Sample Characteristics in Study 2.

Samples	Country	N	Gender %		Age M (SD)
			Male	Female	
1 (original)	United States	120	31.7	67.5	20.28 (1.35)
2 (new)	The Netherlands	44	45.5	54.5	21.59 (1.88)
3 (new)	The Netherlands	43	34.9	62.8	21.55 (2.13)
4 (new)	The Netherlands	33	27.3	72.7	24.45 (2.17)
Combined		240	34.2	65.0	21.32 (2.21)

*Note.* In total, two participants did not report gender; one participant did not report age.

#### Procedure and materials

The new samples were collected using the same procedure and materials from the original O-train experiment (Ren et al., 2016). We conducted the O-train paradigm as a classroom activity at a university in the Netherlands in three occasions. Because all the classes we conducted this activity in were taught in English, all O-train materials were in English. At the beginning of the activity, students were instructed to form triads, sit in a row during a simulated train ride, with one student occupying the center seat (target), and two in the side seats^4^ (source). All “passengers” then received a “train ticket” along with a written script that directed them to act out a scenario. In reality, all triads were randomly assigned to either an inclusion condition or an ostracism condition. In the inclusion condition, side seat participants followed the script to involve the center seat participant in their conversation. In the ostracism condition, side seat participants followed the script to exclude the center seat participant from their conversation. Regardless of the condition, center seat participants were instructed to join the group’s conversation (see Supplemental Materials for specific instructions). The activity lasted approximately 5 min.

Participants then completed the same set of questionnaires from the original study. They first completed a shortened version of the Needs Satisfaction Questionnaire (Ren et al., 2016; Williams, 2009) with 12 items rated on a 5-point scale (1 = *not at all*; 5 = *very much*), assessing their satisfaction for belonging (e.g., “I felt like an outsider”), self-esteem (e.g., “My self-esteem was high”), meaningful existence (e.g., “I felt invisible”), and control (e.g., “I felt I had control over the course of the game”). Items were reverse-coded when necessary and averaged to provide indexes for each need satisfaction (α*_belonging_* = .93, α*_self-esteem_* = .88, α*_meaningful existence_* = .90, α*_control_* = .82). Ostracism manipulations (e.g., O-Train, Cyberball) typically lower these need indexes (Hartgerink et al., 2015; Zadro et al., 2005).

Participants then indicated to what extent they wish they had been alone on the past train ride, and their preferences for the next train ride: ride alone, remain in the same group, or join a new group, respectively, on the same 5-point scale. Following the original paper, we assessed and analyzed these three intentions separately because these intentions may coexist (Sommer & Bernieri, 2015).

As manipulation checks, participants indicated how “ignored” and “excluded” they felt during the activity on the same 5-point scale (Williams, 2009). These two items were averaged to provide a single index (Spearman–Brown coefficient = .96; Eisinga et al., 2013).

### Results

To account for the clustered nature of the data (participants are clustered within triads; triads are clustered within samples), we estimated multilevel models (Raudenbush & Bryk, 2002) with the dummy coded ostracism manipulation (inclusion = 0; ostracism = 1), the assigned role (target = 0; source = 1), and their interaction term as predictors; random-intercepts were estimated for each triad and each sample. We used the R packages lme4 and lmerTest (Bates et al., 2014; Kuznetsova et al., 2015).

Consistently across the models, the interaction term was significant (Table 3). To probe these interaction effects, we conducted simple slope analyses; we focus on these results below (Table 4).

**Table 3.** Multilevel Models (Unstandardized Regression Coefficients) Predicting Each Outcome Variable From the Conditions in Study 2.

Dependent variables	Ostracism		Role		Ostracism * Role	
	B	CI	B	CI	B	CI
MC: being ostracized	2.42***	[2.12, 2.76]	–0.24	[–0.53, 0.03]	–2.48***	[–2.91, –2.10]
Need: belonging	–2.08***	[–2.43, –1.73]	0.35*	[0.08, 0.61]	2.19***	[1.79, 2.58]
Need: self-esteem	–1.55***	[–1.93, –1.17]	0.21	[–0.10, 0.54]	1.15***	[0.67, 1.63]
Need: existence	–1.89***	[–2.26, –1.56]	0.11	[–0.19, 0.41]	1.95***	[1.51, 2.36]
Need: control	–0.74***	[–1.14, –0.36]	0.26	[–0.06, 0.57]	1.49***	[1.06, 1.96]
Wish of solitude	1.67***	[1.14, 2.23]	0.46*	[–0.87, –0.03]	–1.43***	[–2.04, –0.83]
Next: alone	1.45***	[0.86, 2.02]	–0.17	[–0.57, 0.29]	–1.36***	[–2.01, –0.72]
Next: same group	–1.27***	[–1.78, –0.80]	0.26	[–0.16, 0.68]	0.81**	[0.23, 1.43]
Next: new group	0.24	[–0.29, 0.75]	0.23	[–0.19, 0.66]	–0.61*	[–1.22, –0.02]

*Note.* CI = Confidence interval; MC = manipulation check.

*p < .05. **p < .01. *** *p* < .001.

**Table 4.** Simple Slope Analyses (Unstandardized Regression Coefficients) in Study 2.

Dependent variables	Target: ostracism (vs. inclusion)		Source: ostracism (vs. inclusion)	
	B	CI	B	CI
MC: being ostracized	2.42***	[2.12, 2.76]	0.06	[–0.30, 0.18]
Need: belonging	–2.08***	[–2.43, –1.73]	0.11	[–0.13, 0.37]
Need: self-esteem	–1.55***	[–1.93, –1.17]	–0.40**	[–0.67, –0.12]
Need: existence	–1.89***	[–2.26, –1.56]	0.05	[–0.18, 0.28]
Need: control	–0.74***	[–1.14, –0.36]	0.75***	[0.49, 1.04]
Wish of solitude	1.67***	[1.14, 2.23]	0.24	[–0.17, 0.65]
Next: alone	1.45***	[0.86, 2.02]	0.09	[–0.32, 0.53]
Next: same group	–1.27***	[–1.78, –0.80]	–0.46**	[–0.77, –0.13]
Next: new group	0.24	[–0.29, 0.75]	–0.37	[–0.78, 0.02]

*Note.* CI = confidence interval; MC = manipulation check.

*p < .05. **p < .01. *** *p* < .001.

#### Manipulation check

Ostracized targets correctly reported being more ostracized than included targets; no difference was found between sources of ostracism and sources of inclusion.

#### Need satisfaction

We analyzed the four need indexes separately. As expected, compared with included targets, ostracized targets reported lower level of satisfaction with all four needs. The effect of ostracism on sources was less uniform across the needs: Compared with sources of inclusion, sources of ostracism reported lower level of satisfaction with self-esteem, but higher level of satisfaction with control. No difference was found between these two groups in their satisfaction with belonging or meaningful existence.

#### Wish of solitude

Ostracized targets indicated a stronger wish that they had been alone on the “train ride” than included targets. No difference was found between sources of ostracism and sources of inclusion.

#### Next task preference

**Figure 2.**
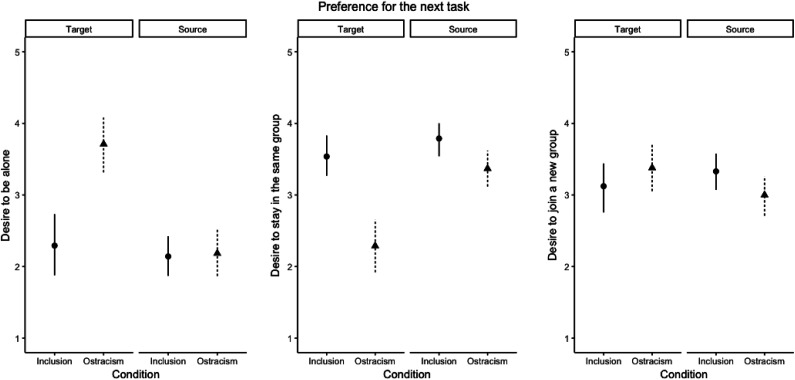
Participants’ preference for the next task as a function of the ostracism manipulation and their assigned role in Study 2. *Note.* The error bars represent confidence intervals.

We analyzed the three items (the desire to be alone, the desire to stay in the same group, and the desire to join a new group) separately. Compared with included targets, ostracized targets indicated a stronger desire to be alone, less desire to stay in the same group, and a similar desire to join a new group. A different pattern of results emerged for sources: Compared with sources of inclusion, sources of ostracism indicated a similar desire to be alone, less desire to stay in the same group, and a similar desire to join a new group (Figure 2).

#### Exploratory analysis: mediation

**Figure 3.**
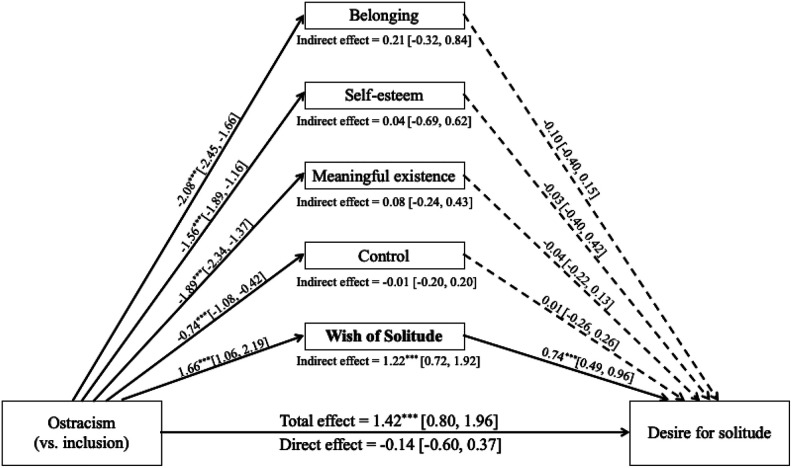
Multiple mediator model testing the indirect effects of ostracism on targets’ desire for solitude in Study 2. *Note.* Coefficients are unstandardized. 95% confidence intervals are in brackets. Solid lines indicate significant paths; dashed lines indicate nonsignificant paths. *p < .05. **p < .01. *** *p* < .001.

In exploring the mechanism that underlies the observed effect of ostracism on targets’ desire for solitude, we conducted a multiple mediation model testing belonging, self-esteem, meaningful existence, control, and wish of solitude each as simultaneous mediators among targets (*n* = 79; Preacher & Hayes, 2008). The four need satisfaction indexes were selected as potential mediators because they are linked with behavioral responses to ostracism (e.g., Wesselmann et al., 2015). Wish of solitude was selected because it reflects one’s consideration of solitude as a possible alternative to their past social interaction experience; this counterfactual thought is likely to direct one’s behavioral intention in a subsequent social interaction (Epstude & Roese, 2008). To reduce the complexity of the model due to clustering, the sample variable (targets are clustered within samples) was dummy coded and entered the model as covariates. We estimated the model using the lavaan package in R (Rosseel, 2012) and requested the bias-corrected and accelerated (BCa) bootstrap intervals based on 5,000 samples. The only significant indirect pathway was through wish of solitude (Figure 3). We will return to these results in the “General Discussion” section.

### Discussion

We replicated the effects of ostracism on the targets from the original O-train experiment. The targets of ostracism, compared with the targets of inclusion, indicated lower need satisfaction, a stronger wish of solitude on the simulated train ride, a stronger desire to be alone in the next activity, less desire to stay in the same group, and a similar desire to join a new group.^5^

Extending beyond the original study, this study revealed the effects of ostracism from the source perspective. Unlike the targets whose satisfaction with four basic needs was uniformly reduced by ostracism, the sources of ostracism (vs. the sources of inclusion) experienced some “benefit” (higher satisfaction with control) and some “cost” (lower satisfaction with self-esteem). This pattern of results fits with the small body of literature that the effect of ostracism on sources is less consistent than the effect on targets (Zadro et al., 2017). The observed “benefit” also fits with previous studies that people experience more control when ostracizing others (Zadro et al., 2017), especially strangers (Nezlek et al., 2015).

In terms of next task preferences, similar to the targets, the sources’ desire to stay in the same group was decreased by ostracism. This finding suggests that both sides of an ostracism event desire to avoid each other immediately after the event even when ostracism was role-played. More importantly, unlike the targets, the sources’ desire for solitude was not affected by ostracism, suggesting that the effect of ostracism on solitude applies to targets only. In brief, ostracism might be stressful and unpleasant for both parties involved; however, only the ones being ostracized, but not the ones ostracizing others desire solitude after the event.

## Study 3

The goal of this study is threefold. Our first goal was to eliminate an alternative explanation to the effect of ostracism on solitude—the feeling of conspicuousness. Following the recommendation from past research (e.g., Williams et al., 2000), here we added a new condition of overinclusion. Overincluded participants are likely to feel conspicuous because they receive excessive attention, but they are not ostracized. We posit that it was the experience of being ostracized, rather than the feeling of conspicuousness, that motivates participants’ solitude desires. Following this reasoning, ostracized participants would report a stronger desire to be alone than included participants; but no difference would emerge between included participants and overincluded participants.

Our second goal was to investigate whether trait extraversion moderates the effect of ostracism on solitude. The original paper predicted and found that the effect of ostracism on solitude was more salient among participants who scored low in extraversion than those scoring high in extraversion. This finding is in line with the conclusion from the literature that introverts are less likely to express that they are in pain or seek social support under stress than extraverts (Phillips & Gatchel, 2000; Swickert et al., 2002). Here, we put the moderation of extraversion to the test again.

Our third and final goal was to replicate and extend the original findings using a different manipulation of ostracism. The original paper used two ostracism paradigms: O-train (Zadro et al., 2005) and Cyberball (Williams et al., 2000). Here, we opted for a recently developed paradigm: Ostracism Online (Wolf et al., 2015), for two reasons. First, this paradigm creates an ostracism experience in a social-media context. Considering the current prevalence of computer-mediated communication, it is ecologically meaningful to replicate the effect in such an environment. Second, Ostracism Online creates an ostracism experience in a group of 12 members, whereas in both paradigms of the original paper, ostracism occurred in a group of 3 (a three-player Cyberball game, or a three-person conversation on a simulated train ride). Considering that group size may influence people’s reactions to ostracism (Sandstrom et al., 2017; Tobin et al., 2018), we chose to test the robustness of the original findings in a larger group.

### Method

#### Participants

Introductory psychology students (*N* = 251) from a large research university in the United States participated in this study for course credits. The sample size was based on the number of students that participated in the study within 3 weeks. Ten participants were not able to complete the survey due to internet malfunction, five participants reported that they were not able to view or like others’ status, and seven participants failed the attention check question. These participants were excluded from data analysis, leaving the final sample size 227 (47.6% male, 52.0% female, one did not report gender; *M*_age_ = 19.18 years, *SD* = 1.79). In this sample, 155 participants identified as Caucasian or White, 40 as Asian or Asian American, 14 as Hispanic, 11 as African Americans, and 7 as “other.”

#### Procedure

Participants were brought into the laboratory and assigned to individual cubicles to complete the study on a computer. Participants first completed a packet of personality measures before taking part in a group introduction activity. In this activity, each participant engaged in an ostensible online group interaction with 11 other participants, who were in fact preprogramed virtual confederates. Before the group interaction, participants were first instructed to prepare a personal profile, containing a nickname, an avatar, and a brief text in which they introduce themselves to the rest of the group. Afterward, participants were ostensibly connected with other online participants. On a webpage that was designed to resemble a social media page, participants’ profile was presented along with other online profiles. Participants were told that they could read and react to each other profiles by clicking a “like” button. The total number of likes was displayed underneath each profile. In reality, participants were randomly assigned to be ostracized, included, or overincluded by the computer-programed confederates. The average number of “likes” received by all the group members was preprogramed to be 5.5; the number of “likes” participants received were preprogramed to be below average (*n* = 1) in the ostracism condition, close to average (*n* = 5) in the inclusion condition, and above average (*n* = 9) in the overinclusion condition. This online interaction lasted for 3 min. After the group activity, participants completed several measures to indicate their experiences during the activity and their preference for the next task. Finally, participants were debriefed and thanked.

### Materials

#### Extraversion

Before Ostracism Online, participants completed the Big Five Inventory (α_Extraversion_ = .90, α_Agreeableness_ = .76, α_Conscientiousness_ = .83, α_Neuroticism_ = .79, α_Openness_ = .79; John & Srivastava, 1999). Following the analytic approach of the original paper, we tested the moderating role of extraversion while including the other four traits as covariates. All five variables were mean-centered before analyzed.

#### Need satisfaction

Same measure from Study 2 (α_belonging_ = .89, α_self-esteem_ = .85, α_meaningful existence_ = .90, α_control_ = .68).

#### Mood

Participants completed a mood measure (Williams, 2009) to indicate how they felt during the group introduction task. The measure included eight items (good, bad, friendly, unfriendly, angry, pleasant, happy, and sad) rated on a 5-point scale (1 = *not at all*; 5 = *extremely*). Items were reverse-coded when necessary and averaged to provide an index for mood, with higher numbers indicating more positivity (α = .91). Ostracism manipulations lower participants’ mood when using this measure (e.g., Williams, 2009).

#### Next task preference

Same measure from Study 2.

#### Manipulation check

Participants completed four manipulation check items. Participants first indicated their agreement to three statements describing their experiences during the group introduction activity (“I was ignored,” “I was excluded,” and “The others liked my description”) on a 5-point scale (1 = *not at all*; 5 = *extremely*). These three items were combined to form an index such that higher number indicates stronger feeling of being ostracized (α = .93). In addition, participants were asked to estimate the number of “likes” they received relative to the rest of the group on a 3-point scale (1 = *under average*, 2 = *about average*, 3 = *above average*; Wolf et al., 2015).

#### Additional measures

Participants also answered a few additional measures^6^ that were unrelated to this report.

### Results

For all analyses (unless otherwise specified), we used multiple linear regression, with dummy coded experimental conditions as predictors (inclusion condition as the reference category; Table 5).

**Table 5.** Regression Models (Unstandardized Regression Coefficient) Predicting Each Outcome Variable From the Conditions in Study 3.

Dependent variables	Ostracism (vs. inclusion)		Overinclusion (vs. inclusion)	
	B	CI	B	CI
MC: feeling ostracized	1.54***	[1.30, 1.78]	*–*0.28*	[–0.51, –0.04]
MC: estimated number of “likes”	–1.11***	[–1.21, –1.01]	0.78***	[0.69, 0.88]
Need: belonging	–1.21***	[–1.47, –0.95]	0.11	[–0.14, 0.36]
Need: self-esteem	–0.88***	[–1.11, –0.64]	0.44***	[0.21, 0.67]
Need: existence	–1.15***	[–1.41, –0.90]	0.03	[–0.22, 0.28]
Need: control	–0.27	[–0.55, 0.01]	0.07	[–0.20, 0.34]
Mood	–0.68***	[–0.88, –0.47]	0.14	[–0.07, 0.34]
Next task: alone	0.64**	[0.23, 1.04]	0.06	[–0.33, 0.45]
Next task: same group	–0.68***	[–1.05, –0.30]	0.15	[–0.21, 0.52]
Next task: new group	–0.29	[–0.65, 0.08]	–0.59**	[–0.95, –0.23]

*Note.* CI = confidence interval; MC = manipulation check.

*p < .05. **p < .01. *** *p* < .001.

#### Manipulation check

Ostracism Online effectively manipulated the feeling of ostracism. Analysis revealed a stepwise pattern of results: ostracized participants accurately reported being more ostracized than included participants; and included participants accurately reported being more ostracized than overincluded participants.

Generally, participants accurately estimated the number of “likes” they received relative to the rest of the group. Specifically, on average, ostracized participants reported that the number of “likes” they received were under average (*M* = 1.04, *SD* = 0.20); included participants reported that the number of “likes” they received were about average (*M* = 2.15, *SD* = 0.40); and overincluded participants reported that the number of “likes” they received were above average (*M* = 2.93, *SD* = 0.25). Analysis revealed a stepwise pattern of results: As intended, ostracized participants’ estimation was lower than included participants and included participants’ estimation was lower than overincluded participants.

#### Need satisfaction

Compared with included participants, ostracized participants reported lower level of satisfaction with belonging, self-esteem, meaningful existence, but not control. Compared with included participants, overincluded participants reported higher level of satisfaction with self-esteem, but not other needs.

#### Mood

Compared with included participants, ostracized participants reported less positive mood. No significant difference was found between included participants and overincluded participants.

#### Next task preference

**Figure 4.**
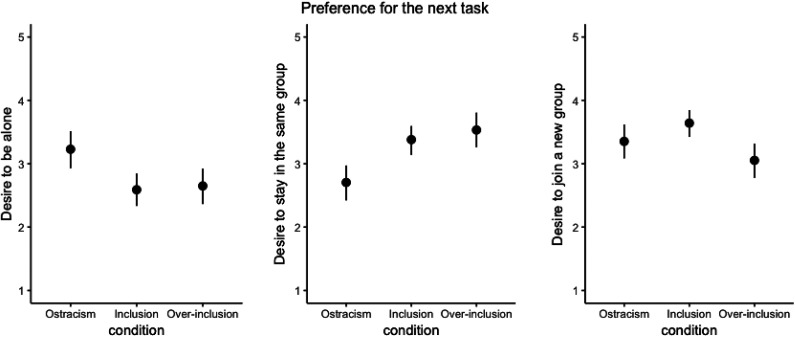
Participants’ preference for the next task as a function of the manipulation in Study 3. *Note.* The error bars represent 95% confidence intervals.

Same as in Study 2, we analyzed the three items (the desire to be alone, the desire to stay in the same group, and the desire to join a new group) separately. The pattern of results replicated Study 2: Compared with included participants, ostracized participants indicated a stronger desire to be alone, less desire to stay in the same group, and a similar desire to join a new group. In contrast, a different pattern of results emerged for overincluded participants: Compared with included participants, they indicated a similar desire to be alone, a similar desire to stay in the same group, but less desire to join a new group (Figure 4).

#### Moderation by extraversion

We further examined the moderating role of extraversion on participants’ preference for solitude. We conducted a multiple regression analysis with two dummy coded condition variables, the extraversion score, their interactions as predictors, and the four other Big Five traits as covariates. Results showed that the only significant effect was the main effect of ostracism, indicating that ostracized participants reported a stronger desire to be alone than included participants (*B* = 0.57, CI = [0.17, 0.96], *p* = .005). No other effects were significant (|*B*|s < 0.41, *p*s > .068).

#### Exploratory analysis: mediation

**Figure 5.**
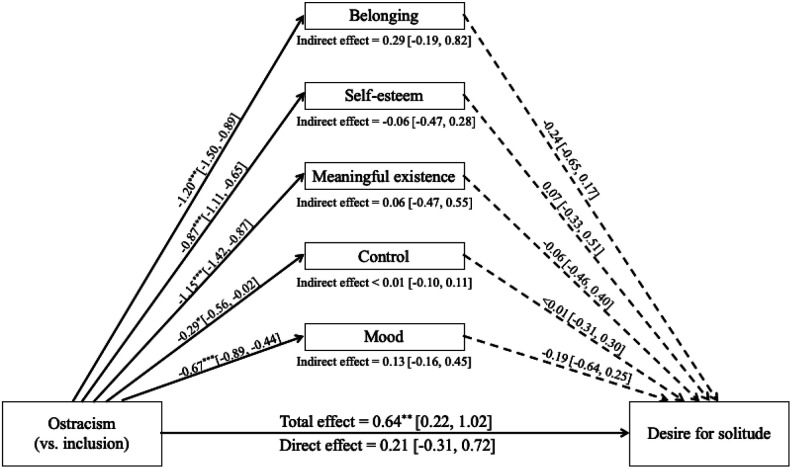
Multiple mediator model testing the indirect effects of ostracism (vs. inclusion) on targets’ desire for solitude in Study 3. *Note.* Coefficients are unstandardized. 95% confidence intervals are in brackets. Solid lines indicate significant paths; dashed lines indicate nonsignificant paths. *p < .05. **p < .01. *** *p* < .001.

Similar to Study 2, in exploring the mechanisms by which ostracism (vs. inclusion) affects solitude seeking, we conducted a multiple mediation model (*n* = 148;^7^ Preacher & Hayes, 2008). Although the indirect effects via the four need satisfaction variables were not significant in Study 2, here we included them again as potential mediators. Mood was selected as an additional potential mediator because ostracism lowers mood (e.g., Williams, 2009) and people may seek solitude to regulate their emotions (Nguyen et al., 2018). Same as in Study 2, we estimated the model using the R package lavaan (Rosseel, 2012) and requested the BCa bootstrap intervals with 5,000 samples. None of the indirect effects were significant (Figure 5). We return to these results in the “General Discussion” section.

### Discussion

We replicated the main effects of ostracism from the original paper. Ostracized participants, compared with included participants, indicated lower need satisfaction,^8^ a less positive mood, a stronger desire to be alone in the next activity, less desire to stay in the same group, and a similar desire to join a new group. However, we failed to replicate the moderating effect of trait extraversion (we return to this finding in General Discussion).

Extending beyond the original paper, two additional findings emerged in this study. First, although both overincluded participants and ostracized participants were likely to feel conspicuous, ostracized participants reported an increased desire to be alone than included participants, whereas overincluded participants did not. This finding helps to eliminate the confounding effect of feeling conspicuous. Second, although overincluded participants accurately recognized that they received excessive attention, indicated by our manipulation checks, they did not view this as a more positive experience than included participants. In fact, except that overinclusion increased the need satisfaction with self-esteem and the desire to stay in the same group, no additional difference was found between these two inclusive conditions. This finding might be counterintuitive, but is in line with past studies which consistently found overinclusion was experienced at a similar level of positivity as inclusion (e.g., Wolf et al., 2015; Van Beest & Williams, 2006; Williams et al., 2000). See Supplemental Materials for further discussion of the comparison between overinclusion and inclusion.

## General Discussion

Ren and colleagues (2016) predicted and found that, in addition to the well-documented prosocial and antisocial responses, solitude seeking is another viable option after being ostracized. Here, we conducted three conceptual replication studies to further test this prediction. Supporting the general conclusion from the original paper, we found that the general experience of being ostracized correlates with a general preference for solitude (Study 1), and experiment manipulated experience of being ostracized leads to a desire for solitude (Studies 2 and 3). These findings were obtained with studies of increased statistical power, data collected in a different cultural context, and a new paradigm of high ecological validity.

The current research extends the original paper in two primary ways. First, we demonstrated that, despite ostracism being unpleasant for all parties involved, only targets have an increased desire for solitude after ostracism but not sources (Study 2). Second, we demonstrated that only being ostracized (vs. included) increased solitude desires but not being overincluded, eliminating the alternative explanation that ostracized participants sought solitude simply because they felt conspicuous (Study 3).

An additional extension beyond the original paper involves exploring the mechanisms by which ostracism triggers targets’ intention to seek solitude (Studies 2 and 3). Although the effect of ostracism on the four needs satisfaction and mood is among the most robust findings in the literature (Hartgerink et al., 2015), none of the indirect effects via these variables were significant in our mediation analyses. This is in contrast to the theoretical and empirical work linking deprived needs with pro- and antisocial responses to ostracism (e.g., Wesselmann et al., 2015). We did, however, find evidence for the indirect effect through wish of solitude in Study 2. This finding is consistent with the work on counterfactual thinking, which has shown that problems (e.g., being ostracized) activate counterfactual thinking (e.g., “I wish I had been alone” in the past social interaction), and counterfactual thinking produces behavioral change (e.g., seeking solitude in a subsequent social interaction; Epstude & Roese, 2008). Critically, this finding is consistent with the idea, which has been put forward by several theorists, that the ostracized seek solitude to minimize the risk of being ostracized again (Richman & Leary, 2009; Sunami et al., 2019a; Wesselmann et al., 2014). We note, however, these mediation results, while informative and interesting, are exploratory and need future confirmatory research.

One finding in the original paper that we did not replicate is the moderating role of extraversion (Study 3). Motivated to better understand this lack of moderation, we conducted simple slope analyses despite nonsignificant interaction terms. Although the effect of overinclusion (vs. inclusion) remained nonsignificant, regardless of participants’ trait extraversion (low: *B* = 0.28, CI = [–0.30, 0.86], *p* = .341; high: *B* = –0.15, CI = [–0.68, 0.39], *p* = .586), the effect of ostracism (vs. inclusion) differed depending on participants’ trait extraversion (low: *B* = 0.91, CI = [0.36, 1.47], *p* = .001; high: *B* = 0.22, CI = [–0.35, 0.79], *p* = .448). Thus, these exploratory analyses offer some support for the moderation of extraversion reported in the original paper: The effect of ostracism on solitude is more prominent for introverts than for extraverts. However, because the evidence is weak, and the analyses are exploratory, future research is needed to clarify the role of extraversion.

In contrast to prior work (e.g., Maner et al., 2007), ostracism did not increase participants’ interest in connecting with a new group (Studies 2 and 3). This finding, although notably diverged from the literature, is consistent with those of the original paper (Ren et al., 2016) and a recent failed replication of Maner et al. (2007; Sunami et al., 2019b). One possible explanation for the mixed evidence is that ostracized participants may be motivated to establish new social connections, but may also feel reluctant to engage in cognitively demanding tasks (Baumeister et al., 2002 but see Juanchich et al., 2018) such as navigating a social interaction with novel partners. Another possible explanation is that participants’ interest in reconnecting with someone else, which was usually measured as the only option in past research (e.g., Maner et al., 2007), was measured alongside with other options in the current research and in Sunami et al. (2019b). We suspect that ostracized participants are highly motivated to look for opportunities to cope with the stress of ostracism, and the availability or salience of a particular response might guide their response (Wesselmann et al., 2015; Schade et al., 2014). For example, when an affiliative response is available or salient to the participants, they would show a greater interest in that option because that is the only opportunity available to cope with ostracism. However, when multiple options are available, they are less likely to be compelled toward a particular course of action (Ren et al., 2016); thus, multiple options would allow researchers a higher chance of observing participants’ natural preference.

The current research also contributes to the growing body of literature on solitude. The vast literature has primarily focused on the negative experiences of being alone (e.g., loneliness; Coplan et al., 2019). Only a few researchers have investigated solitude from a more neutral perspective, providing evidence that solitude could be welcome and enjoyable at times (e.g., Coplan et al., 2019; Nguyen et al., 2018). Despite the growing interest in this topic, when people voluntarily choose to be alone remains poorly understood. Our research contributes to this area of research by uncovering one situational factor that stimulates the motivation for solitude: being ostracized. Furthermore, contrary to one’s intuition that people seek solitude because they are low in extraversion, this state motivation for solitude as a result of ostracism had no clear relation with extraversion (see our discussion above for the inconsistent moderating effect of extraversion).

### Limitations and Future Research

First, although beyond the scope of the current research, we recognize the need and the challenge to organize the various responses to ostracism in a coherent theoretical framework. When and why does ostracism lead to a prosocial response, an antisocial response, or solitude seeking? We suspect the research practice that only providing participants with one possible response might have contributed to our lack of understanding of this question. Future research may provide participants with a list of options that allow for all three responses to systematically study the moderators of ostracism response. In addition to this methodological consideration, we believe that the field of ostracism would benefit from drawing insights from broader literature on interpersonal behavioral tendencies (Van Kleef et al., 2010). For example, it is been speculated that people are more likely to simply “leave the field” when people lack the motivation to change the status quo (Van Kleef et al., 2010). Following this line of reasoning, it is possible that when changing the exclusionary status is not sufficiently rewarding (e.g., being included by strangers online), people are less willing to take on the risk of being ostracized again and consequently avoid social interactions (Richman & Leary, 2009; Sunami et al., 2019a). In contrast, when re-inclusion is highly rewarding (socially: e.g., repairing one’s close relationship; Richman & Leary, 2009; or financially: e.g., being included in a profitable negotiation; Walasek et al., 2019), people should be more willing to take actions, either prosocial or antisocial, to increase their chance of social or financial gains. In the current experiments, ostracism responses were assessed in a low-rewarding context. Future researchers should consider studying participants’ responses in more rewarding situations.

Second, we sampled from independent culture groups (The Netherlands, the United States). Thus, it is unclear whether the effect of ostracism on solitude generalize across cultures. A few studies have shown that more socially interdependent individuals are less affected by ostracism or recover faster than less interdependent individuals (Pfundmair et al., 2015; Ren et al., 2013). One interpretation of these findings is that social support, working as a buffer against the pain of an ostracism episode, is more mentally accessible to highly interdependent individuals (Gardner et al., 2005; Uskul & Over, 2017). Thus, we speculate that ostracism-induced solitude desires might be weaker among more (vs. less) interdependent individuals.

Third, we used samples of young adults. Thus, it is unclear whether the effect of ostracism on solitude generalizes to other age groups. Existing studies that included age as a potential moderator are inconsistent: The impact of ostracism on older adults (vs. younger adults) has been found to be weaker (Charles & Carstensen, 2008; Hawkley et al., 2011), stronger (Cheng & Grühn, 2015), or similar (Löckenhoff et al., 2013). Although it is challenging to conclude based on existing research whether and how age moderates the impact of ostracism, it is well documented that social contacts decline with age. One reason is that older adults are more selective in choosing their social partners and more motivated to avoid potentially negative interactions compared with younger adults (Charles & Carstensen, 2010; Nikitin et al., 2014). Thus, after being ostracized, older participants (vs. younger) might be more likely to move away from social interactions.

Finally, although our research contributes to the growing literature on sources of ostracism, our method of studying the sources has shortcomings. First, sources in our paradigm followed instructions to ostracize; however, sources in real life may have strong motives behind their actions (Sommer & Yoon, 2013; Zadro & Gonsalkorale, 2014), from protecting a group from threatening members, to correcting undesirable behaviors, to removing deviant individuals (Hales et al., 2014). Without these motives, ostracizing can be psychologically costly, incurring negative affect, feelings of guilt and shame, and reduced senses of autonomy and social connection (Gooley et al., 2015; Legate et al., 2013). Subsequently, sources of ostracism are likely to have greater sympathy for the target and engage in compensatory behaviors toward the target (Van Tongeren et al., 2015; Wesselmann et al., 2013). Second, in our study participants ostracized others with a co-source. Co-source can provide a sense of belonging and reduce the sense of responsibility for inflicting harm on targets (Zadro & Gonsalkorale, 2014). Thus, being the sole ostracizer (e.g., in dyadic relationships) may lead to more negative outcomes for the source. Third, our study did not examine personal factors; but sources may react differently based on their personality traits. Past work found that not all sources react the same way: While some sources regret ostracizing others, other sources (e.g., people who lack communication skills, or low in trait self-esteem) frequently resort to ostracism when interpersonal conflicts occur (Zadro et al., 2017). Identifying the ostracizing-related personal factors is a promising avenue of future research.

## Conclusion

People have varied responses to ostracism. A widely accepted conclusion in this area of research has been that people either behave prosocially or antisocially after being ostracized. A recent report (Ren et al., 2016) updated this conclusion by providing empirical evidence to a third option of solitude seeking. Our current program of studies supports this conclusion with additional and stronger evidence. Extending beyond the original paper, our studies further showed that only the experience of being ostracized, but not ostracizing others or the feeling of conspicuousness, triggered the desire for solitude. Exploratory mediation analyses shed light on a mechanism: ostracized targets considered solitude as an appealing alternative to their past experience; this counterfactual thinking oriented them toward solitude to avoid being ostracized again.

## Supplemental Material

Supplemental Material, Ren_Online_Appendix - Seeking Solitude After Being Ostracized: A Replication and Beyond Click here for additional data file. Supplemental Material, Ren_Online_Appendix for Seeking Solitude After Being Ostracized: A Replication and Beyond by Dongning Ren, Eric D. Wesselmann and Ilja van Beest in Personality and Social Psychology Bulletin

Solitude_Supplementary_Materials_2020-04-23 - Seeking Solitude After Being Ostracized: A Replication and Beyond Click here for additional data file. Solitude_Supplementary_Materials_2020-04-23 for Seeking Solitude After Being Ostracized: A Replication and Beyond by Dongning Ren, Eric D. Wesselmann and Ilja van Beest in Personality and Social Psychology Bulletin

## Notes

1. Given the ongoing debates about the specific theoretical and empirical differences between ostracism and those of other forms of social exclusion and rejection, we use the three terms interchangeably in this article.

2. All confidence intervals in this report are 95% confidence intervals.

3. As a direct replication, we followed the analytic approach of the original paper and tested the effect of ostracism on targets using only the newly collected samples. We obtained the same conclusions of the original paper. See Supplemental Materials for details.

4. When the number of students did not allow for only groups of 3, one group in that session took on one additional member. In the combined sample, in total three groups had four students. In these groups, only one student was assigned to be the target.

5. Although the null effect of ostracism on targets’ desire to join a new group is consistent with the original paper (we found this null effect again in Study 3), it is inconsistent with past research which has shown that excluded individuals seek affiliative opportunities (e.g., Maner et al., 2007). We will return to this point in the “General Discussion” section.

6. Before the manipulation, participants completed measures about their preference for solitude and their attachment orientation. After the manipulation, participants answered questions about how they felt after the group introduction task, and their behavioral intentions toward other members.

7. Two participants were excluded from analysis due to missing data on desire for solitude.

8. Ostracized participants (vs. included participants) reported lower need satisfaction with belonging, self-esteem, and meaningful existence, but not with control. This is in contrast to the well-established conclusion that ostracism decreases need satisfaction across all four basic needs (Hartgerink et al., 2015; Williams, 2009). One possible interpretation is that Ostracism Online affords ostracized participants with more control than some other common paradigms in the literature (Schneider et al., 2017). This point is further discussed in Supplemental Materials.
