# Supplementary material for: Seeking Solitude After Being Ostracized: A Replication and Beyond
Source: Pers Soc Psychol Bull. 2020 Jun 9;47(3):426–40. doi: 10.1177/0146167220928238 (PMC7897794; doi:10.1177/0146167220928238)
Supplement: Supplemental Material, Ren_Online_Appendix - Seeking Solitude After Being Ostracized: A Replication and Beyond [file Ren_Online_Appendix.docx]

Table of contents

[Study 1 2](#_Toc17661171)

[Ostracism experiences (English) 2](#_Toc17661172)

[Ostracism experiences (Dutch) 2](#_Toc17661173)

[Preference for solitude (English) 3](#_Toc17661174)

[Preference for solitude (Dutch) 3](#_Toc17661175)

[Demographics (English) 4](#_Toc17661176)

[Demographics (Dutch) 4](#_Toc17661177)

[Study 2 5](#_Toc17661178)

[Manipulation: O-Train 5](#_Toc17661179)

[Need satisfaction 5](#_Toc17661180)

[Wish of Solitude 6](#_Toc17661181)

[Next task preference 6](#_Toc17661182)

[Manipulation check 6](#_Toc17661183)

[Demographics 6](#_Toc17661184)

[Study 3 7](#_Toc17661185)

[Manipulation: Ostracism Online 7](#_Toc17661186)

[Big Five Inventory 7](#_Toc17661187)

[Need satisfaction 9](#_Toc17661188)

[Mood 9](#_Toc17661189)

[Next task preference 9](#_Toc17661190)

[Manipulation check 10](#_Toc17661191)

[Demographics 10](#_Toc17661192)

# Study 1

## Ostracism experiences (English)

For each of the following statements, please consider your personal feelings. Determine how often, in general, the following experiences happen to you. Just give your gut response. Use the sale provided:

| 1 | 2 | 3 | 4 | 5 | 6 | 7 |
| --- | --- | --- | --- | --- | --- | --- |
| Hardly ever |  |  | Sometimes |  |  | Almost always |

In general, others leave me out of their group.

In general, others keep me out-of-the-loop on information.

In general, others treat me as if I am invisible.

In general, others give me the cold shoulder treatment.

In general, others physically turn their backs to me when in my presence.

In general, others treat me as if I’m in solitary confinement.

In general, others do not look at me when I’m in their presence.

In general, others ignore me during their conversation.

## Ostracism experiences (Dutch)

Denk bij elk van de volgende stellingen aan je eigen gevoelens. Geef aan hoe vaak, over het algemeen, je de volgende ervaringen hebt. Geef gewoon je eerste intuïtieve reactie.

| 1 | 2 | 3 | 4 | 5 | 6 | 7 |
| --- | --- | --- | --- | --- | --- | --- |
| Bijna nooit |  |  | Soms |  |  | Bijna altijd |

In het algemeen houden anderen me buiten hun groep.

In het algemeen weerhouden anderen informatie van me.

In het algemeen behandelen anderen me alsof ik onzichtbaar ben.

In het algemeen keren anderen mij de rug toe.

In het algemeen keren mensen mij letterlijk de rug toe als ik aanwezig ben.

In het algemeen behandelen anderen me alsof ik in eenzame opsluiting zit.

In het algemeen kijken anderen niet naar me als ik aanwezig ben.

In het algemeen negeren anderen me tijdens hun gesprek.

## Preference for solitude (English)

To what extent does each of the following statements apply to you? Please respond using a 7-point scale:

| 1 | 2 | 3 | 4 | 5 | 6 | 7 |
| --- | --- | --- | --- | --- | --- | --- |
| Not at all |  |  |  |  |  | Very much |

I do not like being alone.

Being apart from other people for long periods of time does not bother me.

I enjoy the pleasure of solitude.

I see myself as a loner.

The perfect weekend is spent alone.

I prefer spending Friday night alone rather than being with others.

The perfect vacation would be with lots of people around.

My dream vacation is to be alone with no connections to others.

With free time, I prefer to be with others.

I feel I can think clearer when alone.

Solitude helps me center myself.

I do not understand people who choose to be alone.

I need time each day alone to collect my thoughts.

Spending time alone enhances my day.

I need time alone each day.

Being with others for extended amounts of time becomes unbearable.

## Preference for solitude (Dutch)

Geef aan in hoeverre je het eens of oneens bent met de volgende stellingen op een schaal:

| 1 | 2 | 3 | 4 | 5 | 6 | 7 |
| --- | --- | --- | --- | --- | --- | --- |
| helemaal niet |  |  |  |  |  | heel erg |

Ik hou er niet van om alleen te zijn.

Ik vind het niet erg om voor een lange periode gescheiden te zijn van andere mensen.

Ik vind het fijn om alleen te zijn.

Ik zie mezelf als een einzelgänger.

Het perfecte weekend is een weekend alleen.

Ik ben liever een vrijdagavond alleen dan met anderen.

De perfecte vakantie is met veel mensen om me heen.

Mijn droomvakantie is om alleen te zijn zonder contact met anderen.

In mijn vrije tijd ben ik het liefst met andere mensen.

Ik heb het gevoel dat ik helderder kan denken als ik alleen ben.

Alleen zijn helpt me om mijn hoofd leeg te maken.

Ik snap niet waarom mensen ervoor kiezen om alleen te zijn.

Ik heb elke dag tijd nodig om alleen te zijn, zodat ik mijn gedachten op een rijtje kan zetten.

Tijd alleen doorbrengen maakt mijn dag beter.

Ik heb elke dag tijd alleen nodig.

Langere tijd met anderen zijn wordt onverdraaglijk.

## Demographics (English)

What is your Gender?

1. Male
2. Female
3. Other/no answer

What is your age?

15 or younger, 16, 17…63, 64, 65 or older

What is your nationality? ____

## Demographics (Dutch)

Wat is je geslacht?

1. Man
2. Vrouw
3. Anders/Geen antwoord

Wat is je leeftijd?

15 of jonger, 16, 17…63, 64, 65 of ouder

Wat is je nationaliteit? ____

# Study 2

## Manipulation: O-Train

Instruction for Sources of Inclusion:

“You are sitting with a good friend (the other “S”), but between you both is a classmate (the one with a “T” ticket). Even though neither you nor your friend were invited to a party that T gave last weekend, you decide not to make a big deal out of it. As the train pulls away from the platform, T starts to talk with you, and you and your friend pay attention to T and involve him or her in conversations. After the train pulls into the first station, the whistle blows again. Now, you, your friend (the other S) and T continue talking, discussing anything and everything.”

Instruction for Sources of Ostracism:

“You are sitting with a good friend (the other “S”), but between you both is a classmate (the one with a “T” ticket). You and your friends decide to give T the cold shoulder, because neither of you were invited to a party that T gave last weekend. As the train pulls away from the platform, T starts to talk with you, but you and your friend give T very brief responses. After the train pulls into the first station, the whistle blows again. Now, you and your friend talk ONLY to each other.”

Instruction for Targets of Inclusion and Ostracism:

“You are sitting in between two classmates. You know each of them fairly well, but you also know that they are close friends. Actually, you are a little bit anxious about sitting with them. You are not sure whether they feel ok with that you didn’t invite them to a party you gave last weekend. As the train pulls away from the platform, you start to talk to them. After the train pulls into the first station, the whistle blows again. Continue trying to have a conversation with your classmates.”

## Need satisfaction

How did you feel during the train ride?

| 1 | 2 | 3 | 4 | 5 |
| --- | --- | --- | --- | --- |
| Not at all |  |  |  | Very much |

Belonging I felt disconnected.

I felt rejected.

I felt like an outsider.

Self-esteem I felt good about myself.

My self-esteem was high.

I felt liked.

Meaningful existence I felt invisible.

I felt meaningless.

I felt non-existent.

Control I felt powerful.

I felt I had control over the course of the interaction.

I felt superior.

## Wish of Solitude

Please rate the following statements regarding your thoughts while you were on this train ride. To what extent does each statement apply to you?

| 1 | 2 | 3 | 4 | 5 |
| --- | --- | --- | --- | --- |
| Not at all |  |  |  | Very much |

I wish I had sat by myself on this train ride.

## Next task preference

To what extent does each statement apply to you?

| 1 | 2 | 3 | 4 | 5 |
| --- | --- | --- | --- | --- |
| Not at all |  |  |  | Very much |

I would like to sit by myself on the next train ride.

I would like to join the two people I just interacted with for another train ride.

I would like to join some other people I haven't interacted with for another train ride.

## Manipulation check

To what extent does each statement apply to you?

| 1 | 2 | 3 | 4 | 5 |
| --- | --- | --- | --- | --- |
| Not at all |  |  |  | Very much |

I felt ignored.

I felt excluded.

## Demographics

Age ___ Gender: Male Female

Did you know the other two people in your group before this activity?

A. Yes, both of them. B. Yes, one of them. C. No, neither of them.

# Study 3

## Manipulation: Ostracism Online

Welcome to this study

You will complete a number of simple tasks, together with other people, with whom you will be connected via the internet. First, all participants in the study will select personal "avatars" and write short texts to introduce themselves.

Before beginning the tasks, you will spend 3 minutes with the other people. During this time, you will be able to read and react to each-other's brief introductions.

Specific instructions will follow.

Thank you!

Please enter your name

Could be your first name, nickname, or initials.

Please select an avatar

This avatar will represent you during the group task, but will not be linked to any of your responses. Your choice of avatar will not be recorded or analyzed.

Please introduce yourself

Please write a paragraph in which you introduce yourself to the rest of the group.

Write something you would like to tell about yourself - anything you want to share.

Group Introduction

Soon after connecting, you should be able to see the other people's descriptions.

You can read and react to the short introduction they wrote about themselves, by

clicking a "like" button, similar to the "like" button on Facebook ("favorite" on Twitter,

♥ in Tumblr and Instagram, etc.)

Even though your interaction is minimal, please try to form an impression of the

people in the group. Try to imagine them in real life - how they might look

or sound, what kind of people they are, how you would get along with them.

The task will last 3 minutes and is very important to the study, so please be attentive,

without switching pages, or doing unrelated tasks. Questions about the other people

might follow.

If these instructions are clear, you can proceed to log in.

## Big Five Inventory

Here are a number of characteristics that may or may not apply to you. For example, do you agree that you are someone who likes to spend time with others? Please choose a number for each statement to indicate the extent to which you agree or disagree with that statement.

| 1 | 2 | 3 | 4 | 5 |
| --- | --- | --- | --- | --- |
| Disagree  Strongly | Disagree  a little | Neither agree  nor disagree | Agree  a little | Agree  strongly |

I am someone who…
1.     Is talkative
2.     Tends to find fault with others
3.     Does a thorough job
4.     Is depressed, blue
5.     Is original, comes up with new ideas
6.     Is reserved
7.     Is helpful and unselfish with others
8.     Can be somewhat careless
9.     Is relaxed, handles stress well. 
10.  Is curious about many different things
11.   Is full of energy
12.   Starts quarrels with others
13.   Is a reliable worker
14.   Can be tense
15.   Is ingenious, a deep thinker
16.   Generates a lot of enthusiasm
17.   Has a forgiving nature
18.   Tends to be disorganized
19.   Worries a lot
20.   Has an active imagination
21.   Tends to be quiet
22.   Is generally trusting
23.   Tends to be lazy
24.   Is emotionally stable, not easily upset
25.   Is inventive
26.   Has an assertive personality
27.   Can be cold and aloof
28.   Perseveres until the task is finished
29.   Can be moody
30.   Values artistic, aesthetic experiences
31.   Is sometimes shy, inhibited
32.   Is considerate and kind to almost everyone
33.   Does things efficiently
34.   Remains calm in tense situations
35.   Prefers work that is routine
36.   Is outgoing, sociable
37.   Is sometimes rude to others
38.   Makes plans and follows through with them
39.   Gets nervous easily
40.   Likes to reflect, play with ideas
41.   Has few artistic interests
42.   Likes to cooperate with others
43.   Is easily distracted
44.   Is sophisticated in art, music, or literature

## Need satisfaction

*For each question, please click the number that best represents the feelings you were experiencing during the group introduction activity*.

| 1 | 2 | 3 | 4 | 5 |
| --- | --- | --- | --- | --- |
| Not at all |  |  |  | Extremely |

Belonging I felt disconnected.

I felt rejected.

I felt like an outsider.

Self-esteem I felt good about myself.

My self-esteem was high.

I felt liked.

Meaningful existence I felt invisible.

I felt meaningless.

I felt non-existent.

Control I felt powerful.

I felt I had control over the course of the interaction.

I felt superior.

## Mood

*Please click the number that best represents the feelings you were experiencing during the group introduction activity.*

| 1 | 2 | 3 | 4 | 5 |
| --- | --- | --- | --- | --- |
| Not at all |  |  |  | Extremely |

Good

Bad

Friendly

Unfriendly

Angry

Pleasant

Happy

Sad

## Next task preference

Next, you are going to complete another task. You can work on this task by yourself or join other online participants. Please answer the following questions to indicate your preference.

| 1 | 2 | 3 | 4 | 5 |
| --- | --- | --- | --- | --- |
| Not at all |  |  |  | Very much |

I’d like to work on the next task by myself.

I’d like to join the group with whom I just interacted.

I’d like to join some other participants with whom I haven’t interacted.

## Manipulation check

You interacted with a group of participants online earlier. For the next questions, please choose the number that best represents the *thoughts*you were experiencing DURING the group introduction activity.

| 1 | 2 | 3 | 4 | 5 |
| --- | --- | --- | --- | --- |
| Not at all |  |  |  | Extremely |

I was ignored.

I was excluded.

The others liked my description.

Considering that there was some average number of "likes" in the introduction task (for example, around 5), how would you consider the number of "likes" you received?

| 1 | 2 | 3 |
| --- | --- | --- |
| under average | about average | above average |

## Demographics

Age ____

Please choose your gender: Male Female

What is your race/ethnicity?

1. African American
2. Asian/Asian American
3. Caucasian/white
4. Hispanic
5. Other
