# Supplementary material for: Seeking Solitude After Being Ostracized: A Replication and Beyond
Source: Pers Soc Psychol Bull. 2020 Jun 9;47(3):426–40. doi: 10.1177/0146167220928238 (PMC7897794; doi:10.1177/0146167220928238)
Supplement: Solitude_Supplementary_Materials_2020-04-23 - Seeking Solitude After Being Ostracized: A Replication and Beyond [file Solitude_Supplementary_Materials_2020-04-23.docx]

**Supplementary Materials**

[Ostracism experiences (Dutch) in Study 1 1](#_Toc33270980)

[Preference for solitude (Dutch) in Study 1 1](#_Toc33270981)

[O-Train instructions in Study 2 2](#_Toc33270982)

[A direct replication of the original O-train experiment 3](#_Toc33270983)

[The null effect of ostracism on the need satisfaction with control in Study 3 4](#_Toc33270984)

[Comparing overinclusion vs. inclusion in Study 3 5](#_Toc33270985)

# Ostracism experiences (Dutch) in Study 1^[[1]](#footnote-1)^

Denk bij elk van de volgende stellingen aan je eigen gevoelens. Geef aan hoe vaak, over het algemeen, je de volgende ervaringen hebt. Geef gewoon je eerste intuïtieve reactie.

| 1 | 2 | 3 | 4 | 5 | 6 | 7 |
| --- | --- | --- | --- | --- | --- | --- |
| Bijna nooit |  |  | Soms |  |  | Bijna altijd |

- In het algemeen houden anderen me buiten hun groep.
- In het algemeen weerhouden anderen informatie van me.
- In het algemeen behandelen anderen me alsof ik onzichtbaar ben.
- In het algemeen keren anderen mij de rug toe.
- In het algemeen keren mensen mij letterlijk de rug toe als ik aanwezig ben.
- In het algemeen behandelen anderen me alsof ik in eenzame opsluiting zit.
- In het algemeen kijken anderen niet naar me als ik aanwezig ben.

In het algemeen negeren anderen me tijdens hun gesprek.

# Preference for solitude (Dutch) in Study 1

Geef aan in hoeverre je het eens of oneens bent met de volgende stellingen op een schaal:

| 1 | 2 | 3 | 4 | 5 | 6 | 7 |
| --- | --- | --- | --- | --- | --- | --- |
| helemaal niet |  |  |  |  |  | heel erg |

- Ik hou er niet van om alleen te zijn.
- Ik vind het niet erg om voor een lange periode gescheiden te zijn van andere mensen.
- Ik vind het fijn om alleen te zijn.
- Ik zie mezelf als een einzelgänger.
- Het perfecte weekend is een weekend alleen.
- Ik ben liever een vrijdagavond alleen dan met anderen.
- De perfecte vakantie is met veel mensen om me heen.
- Mijn droomvakantie is om alleen te zijn zonder contact met anderen.
- In mijn vrije tijd ben ik het liefst met andere mensen.
- Ik heb het gevoel dat ik helderder kan denken als ik alleen ben.
- Alleen zijn helpt me om mijn hoofd leeg te maken.
- Ik snap niet waarom mensen ervoor kiezen om alleen te zijn.
- Ik heb elke dag tijd nodig om alleen te zijn, zodat ik mijn gedachten op een rijtje kan zetten.
- Tijd alleen doorbrengen maakt mijn dag beter.
- Ik heb elke dag tijd alleen nodig.
- Langere tijd met anderen zijn wordt onverdraaglijk.

# O-Train instructions in Study 2

**Instruction for Sources of Inclusion:** “You are sitting with a good friend (the other “S”), but between you both is a classmate (the one with a “T” ticket). Even though neither you nor your friend were invited to a party that T gave last weekend, you decide not to make a big deal out of it. As the train pulls away from the platform, T starts to talk with you, and you and your friend pay attention to T and involve him or her in conversations. After the train pulls into the first station, the whistle blows again. Now, you, your friend (the other S) and T continue talking, discussing anything and everything.”

**Instruction for Sources of Ostracism:** “You are sitting with a good friend (the other “S”), but between you both is a classmate (the one with a “T” ticket). You and your friends decide to give T the cold shoulder, because neither of you were invited to a party that T gave last weekend. As the train pulls away from the platform, T starts to talk with you, but you and your friend give T very brief responses. After the train pulls into the first station, the whistle blows again. Now, you and your friend talk ONLY to each other.”

**Instruction for Targets of Inclusion and Ostracism:** “You are sitting in between two classmates. You know each of them fairly well, but you also know that they are close friends. Actually, you are a little bit anxious about sitting with them. You are not sure whether they feel ok with that you didn’t invite them to a party you gave last weekend. As the train pulls away from the platform, you start to talk to them. After the train pulls into the first station, the whistle blows again. Continue trying to have a conversation with your classmates.”

# A direct replication of the original O-train experiment

Do the findings of the original O-train experiment replicate in our newly collected samples? Following the analytic approach of the original study, we focused on the targets. To account for the clustered nature of the data (participants are clustered within samples), we estimated multilevel models (Raudenbush & Bryk, 2002) with the dummy coded ostracism manipulation (inclusion = 0; ostracism = 1) as predictor; random-intercepts were estimated for each sample. We used the R packages lme4 and lmerTest (Bates et al, 2014; Kuznetsova et al, 2015).

We obtained the exact same conclusions as the original study: ostracized participants reported a stronger feeling of ostracism and lower need satisfaction than included participants. More importantly, they reported they wish they had been alone; for the next “ride”, they reported a stronger desire to be alone, and less desire to stay in the same group. No evidence suggests that their desire to join a new group differed from that of included participants (see Supplementary Table 1).

Supplementary Table 1.

Multilevel models (unstandardized regression coefficients) predicting each outcome variable from the conditions in Study 2 among the targets (new samples; *N* = 39).

| Dependent variables | Ostracism (vs. Inclusion) | |
| --- | --- | --- |
|  | *B* | *CI* |
| Manipulation Check: being ostracized | 2.26^***^ | [1.58, 2.90] |
| Need: belonging | -1.7^***^ | [-2.31, -1.08] |
| Need: self-esteem | -1.22^***^ | [-1.77, -0.65] |
| Need: existence | -1.97^***^ | [-2.56, -1.39] |
| Need: control | -0.81^**^ | [-1.28, -0.31] |
| Wish of solitude | 1.24^**^ | [0.31, 2.05] |
| Next: alone | 1.03^*^ | [0.14, 2.01] |
| Next: same group | -0.96^**^ | [-1.59, -0.41] |
| Next: new group | 0.47 | [-0.28, 1.13] |

*Note.* ^***^ *p* < .001; ^**^ *p* < .01; ^*^ *p* < .05.

# The null effect of ostracism on the need satisfaction with control in Study 3

One unexpected finding is the null effect of ostracism (vs. inclusion) on satisfaction with need for control. Typically, ostracism decreases need satisfaction with all four basic needs including the need for control (Hartgerink et al., 2015; Williams, 2009). However, the few studies that used Ostracism Online produced inconsistent effect of ostracism on control (Schneider, Zwillich, Bindl, Hopp, Reich, & Vorderer, 2017; Wolf et al., 2015). One possible interpretation is that the unique feature of Ostracism Online affords ostracized participants with more control than some other common paradigms in the literature. For example, in O-train, ostracized participants cannot take any actions once they are excluded from the conversation. Similarly, in a Cyberball game, ostracized participants cannot take any actions once they stopped receiving any ball toss. In these paradigms, ostracized participants lack the ability to engage the source of ostracism or take any action in any way at all. However, in the current Ostracism Online paradigm, ostracized participants still have the opportunity to read and “like” other people’s texts, which might have given them some sense of control (Schneider et al, 2017). As a result, the effect size of ostracism on control might have been smaller in Ostracism Online than other paradigms and we might not have had enough power to detect this effect. Indeed, based on our data and the data from the original paper, while included participants reported comparable level of control satisfaction in these three paradigms (Ostracism Online: *M*_study 3_ = 2.49; O-train: *M*_study 2_ = 2.40, *M*_original study 3_ = 2.32; Cyberball: *M*_original study 2_ = 2.63, *M*_original study 4_ = 2.43), ostracized participants seem to have reported more satisfaction with control in this paradigm than those in other paradigms (Ostracism Online: *M*_study 3_ = 2.22; O-train: *M*_study 2_ = 1.66, *M*_original study 3_ = 1.63; Cyberball: *M*_original study 2_ = 1.52, *M*_original study 4_ = 1.63).

# Comparing overinclusion vs. inclusion in Study 3

Intuitively, overinclusion may be a more positive experience than inclusion. However, it has been consistently shown that overinclusion was not a more positive experience than inclusion (Kawamoto et al., 2012; van Beest & Williams, 2006; Van Beest, Williams, & Van Dijk, 2011; Williams, Cheung, & Choi, 2000; Wolf, et al., 2015). This finding replicates across different paradigms (Cyberball, Ostracism Online) and various outcome variables (need satisfaction, mood, social pain).

Why doesn’t overinclusion bring additional benefits beyond inclusion? One possible reason is that overinclusion leads to feeling of conspicuousness (Kawamoto et al., 2012), which people may find uncomfortable. Another reason could be inclusion satiates the need to belong (Dewall, Baumeister, & Vohs, 2008) and people do not derive happiness or satisfaction from additional social attention beyond inclusion. Perhaps the economic law of diminishing marginal utility (Gossen, 1854/1983) applies to social attention as well: as social attention increases, the additional happiness and satisfaction derived from each additional unit declines. This notion received support from a recent study which showed social contact beyond a certain point is no longer associated with better subjective well-being (Kushlev, Heintzelman, Oishi, & Diener, 2018).

**References**

Bates, D., Maechler, M., Bolker, B., & Walker, S. (2014). lme4: Linear mixed-effects models using Eigen and S4. Retrieved from <https://cran.r-project.org/package=lme4>

DeWall, C. N., Baumeister, R. F., & Vohs, K. D. (2008). Satiated with belongingness? Effects of acceptance, rejection, and task framing on self-regulatory performance. *Journal of Personality and Social Psychology, 95,* 1367.

Gossen, H. H. (1854/1983). The laws of human relations and the rules of human action derived therefrom. Cambridge, MA: MIT Press.

Hartgerink, C. H., Van Beest, I., Wicherts, J. M., & Williams, K. D. (2015). The ordinal effects of ostracism: A meta-analysis of 120 Cyberball studies. *PloS One, 10*, e0127002.

Kawamoto, T., Onoda, K., Nakashima, K., Nittono, H., Yamaguchi, S., & Ura, M. (2012). Is dorsal anterior cingulate cortex activation in response to social exclusion due to expectancy violation? An fMRI study. *Frontiers in Evolutionary Neuroscience, 4,* 11.

Kushlev, K., Heintzelman, S. J., Oishi, S., & Diener, E. (2018). The declining marginal utility of social time for subjective well-being. Journal of Research in Personality, 74, 124-140.

Kuznetsova, A., Brockhoff, P. B., & Christensen, R. H. B. (2015). lmerTest: Tests for random and fixed effects for linear mixed effect models (lmer objects of lme4 package). Retrieved from <https://cran.r-project.org/package=lmerTest>

Raudenbush, S. W., & Bryk, A. S. (2002). *Hierarchical linear models: Applications and data analysis methods*. Thousand Oaks, CA: Sage Publications, Inc.

Schneider, F. M., Zwillich, B., Bindl, M. J., Hopp, F. R., Reich, S., & Vorderer, P. (2017). Social media ostracism: The effects of being excluded online. *Computers in Human Behavior, 73,* 385-393.

Van Beest, I., & Williams, K. D. (2006). When inclusion costs and ostracism pays, ostracism still hurts. *Journal of Personality and Social Psychology, 91*, 918-928.

Van Beest, I., Williams, K. D., & Van Dijk, E. (2011). Cyberbomb: Effects of being ostracized from a death game. *Group Processes and Intergroup Relations, 14,* 581–596.

Williams, K. D. (2009). Ostracism: Effects of being excluded and ignored. *Advances in Experimental Social Psychology, 41*, 275-314.

Williams, K. D., Cheung, C. K., & Choi, W. (2000). Cyberostracism: Effects of being ignored over the Internet. *Journal of Personality and Social Psychology, 79*, 748-762.

Wolf, W., Levordashka, A., Ruff, J. R., Kraaijeveld, S., Lueckmann, J. M., & Williams, K. D. (2015). Ostracism Online: A social media ostracism paradigm. *Behavior Research Methods, 47,* 361-373.

1. See our OSF page for the English versions of the ostracism experience scale and the preference for solitude scale: [https://osf.io/9rvb3/](https://osf.io/9rvb3/?view_only=d633d74baeef4f26ba41de98949199e1) [↑](#footnote-ref-1)
